# Empagliflozin in the treatment of heart failure and type 2 diabetes mellitus: Evidence from several large clinical trials

**DOI:** 10.7150/ijms.72772

**Published:** 2022-06-21

**Authors:** Bo Liang, Ning Gu

**Affiliations:** 1 Nanjing University of Chinese Medicine, Nanjing, China.; 2 Department of Cardiology, The Second Affiliated Hospital, School of Medicine, Zhejiang University, Hangzhou, China.; 3 Nanjing Hospital of Chinese Medicine Affiliated to Nanjing University of Chinese Medicine, Nanjing, China.

**Keywords:** empagliflozin, SGLT2i, heart failure, type 2 diabetes mellitus

## Abstract

Heart failure coexists with type 2 diabetes mellitus, which seriously affects the clinical treatment and prognosis. At present, the treatment for patients with established heart failure and type 2 diabetes mellitus is usually combined with two treatment strategies for heart failure and type 2 diabetes mellitus. Recently, increasing studies showed that empagliflozin, a sodium-glucose co-transporter-2 inhibitor, has a positive effect on the treatment of patients with established heart failure and type 2 diabetes mellitus. Here, we summarize the latest and current understanding of the management for patients with established heart failure and type 2 diabetes mellitus and further present contemporary treatment options, sodium-glucose co-transporter-2 inhibitor, for these particular populations.

## Introduction

Heart failure (HF) and type 2 diabetes mellitus (T2DM) are common medical diseases 1, 2, especially among the elderly. Two diseases are usually diagnosed in the same patient at the same time, which undoubtedly increases the difficulty of clinical management and worsens the prognosis of patients 3, 4. The clinical condition of patients with T2DM complicated with HF, both HF with reduced ejection fraction (HFrEF) and HF with preserved ejection fraction (HFpEF), is worse than that of patients with HF without T2DM, and all-cause mortality and cardiovascular mortality are higher 5-7. In another word, T2DM exacerbates the prognosis of HF. Moreover, studies show that HF is an independent predictor of clinical prognosis, whether fatal or nonfatal, in diabetic patients 8-11. The interaction between HF and T2DM makes the prognosis of patients more unsatisfactory.

When a patient is diagnosed with HF and T2DM at the same time, our current understanding is that, in general, all HF treatments are similarly effective irrespective of T2DM. In recent years, many hypoglycemic drugs, such as sodium-glucose co-transporter-2 inhibitors (SGLT-2is), have emerged in the treatment of HF. Although the potential benefits and risks of SGLT-2is are unclear 12, SGLT-2is significantly reduce cardiovascular events, including hospitalization for HF and all-cause hospitalization or death 13-17. Scandinavian register-based cohort study indicated that SGLT-2i lowers HF risk compared with dipeptidyl peptidase-4 inhibitor 18, another glucose-lowering drug. This benefit from SGLT-2i may contribute to the upregulation of the renin-angiotensin-aldosterone system 19. Empagliflozin, an SGLT2i, significantly reduces hospitalization for HF in patients with established cardiovascular disease or at risk of cardiovascular disease and improves cardiac function in HFrEF independent of loading conditions 20. The latest guideline recommended that empagliflozin should be considered in patients with T2DM and either established cardiovascular disease or at high cardiovascular risk to delay or prevent the onset of HF or prolong life 21. A meta-analysis also indicated that compared with placebo, empagliflozin reduced all-cause and cardiovascular mortality independent of baseline risk 22. Of note, few trials supported the recommendation where the presence of HF at baseline was well-characterized or phenotyped, indicating that evidence supporting empagliflozin for patients with HF and T2DM is still insufficient. Here, we critically review and distill contemporary evidence of empagliflozin for treating established HF and T2DM, with a view to provide a more systematic, comprehensive and rational understanding of this treatment option.

### EMPA-REG OUTCOME

EMPA-REG OUTCOME was conducted to assess the effect of empagliflozin on cardiovascular events in adults with T2DM at high cardiovascular risk 23. The primary outcome was cardiovascular mortality, nonfatal myocardial infarction, or nonfatal stroke, and the key secondary composite outcome was the primary outcome plus hospitalization for unstable angina. After a median observation time of 3.1 years, patients in the empagliflozin group reduced the primary outcome and the key secondary composite outcome compared with those in the placebo group. Moreover, empagliflozin significantly lowered hospitalization for HF, cardiovascular mortality, and all-cause mortality than placebo, whereas reduced nonfatal myocardial infarction and nonfatal stroke with no significance to placebo. In short, EMPA-REG OUTCOME demonstrated that empagliflozin reduced hospitalization for HF risk on top of the standard of care in patients with T2DM and established cardiovascular disease 24. The post hoc evaluation showed that the changes in hematocrit and hemoglobin were the most important mediators of the reduction in hospitalization for HF and death from HF 25.

### EMPRISE

EMPRISE used real-world data from three databases in the USA to evaluate the effectiveness, safety, and impact on healthcare utilization of empagliflozin 26. The interim analysis of EMPRISE evaluated the impact of empagliflozin on hospitalization for HF and compared it with sitagliptin, a dipeptidyl peptidase-4 inhibitor, which has proven to have a neutral impact on hospitalization for HF 27. Among included patients, only approximately 5% had existing HF. Over a mean follow-up of 5.3 months, the initiation of empagliflozin decreased hospitalization for HF compared with the initiation of sitagliptin. Moreover, some patients with no history of HF developed HF during the follow-up, and empagliflozin reduced hospitalization for HF regardless of the history of HF 26.

### EMPEROR-Reduced

EMPEROR-Reduced determined whether empagliflozin significantly increased the clinical benefit of HFrEF patients 28. EMPEROR-Reduced indicated that empagliflozin significantly lowered hospitalization for HF and cardiovascular mortality than placebo, with or without T2DM 29.

### EMPEROR-Preserved

EMPEROR-Preserved enrolled 5988 patients with HFpEF, with and without T2DM. EMPEROR-Preserved indicated that empagliflozin reduced hospitalization for HF and cardiovascular mortality 30.

### EMPA-TROPISM

EMPA-TROPISM compared the efficacy of and safety of empagliflozin in non-diabetic HFrEF (left ventricular ejection fraction < 50%) patients 31. Empagliflozin significantly improved left ventricular end-diastolic and end-systolic volumes decreased left ventricular mass, increased left ventricular ejection fraction, enhanced functional capacity as per peak O_2_ consumption oxygen uptake efficiency slope, and 6-min walk test, and improved quality of life as per the Kansas City Cardiomyopathy Questionnaire-12 when compared with placebo 32. EMPA-TROPISM also demonstrated that empagliflozin regressed left ventricular interstitial fibrosis, improved aortic stiffness, regressed epicardial adipose tissue, and exerted an anti-inflammatory effect 33, which provides a new clue for the mechanism of empagliflozin.

## Discussion

EMPA-REG OUTCOME, EMPRISE, EMPEROR-Reduced, and EMPEROR-Preserved all showed that empagliflozin reduces the risk of hospitalization for HF among specific individuals. EMPA-TROPISM provides something new about the mechanism of empagliflozin. Nevertheless, these patients included in the trials did not have an established HF combined with T2DM or the degree of HF combined with T2DM was low at baseline, indicating the evidence of the clinical benefits of empagliflozin on HF patients combined with T2DM are still not sufficient, therefore, long-term clinical exploration including both HF with T2DM patients is needed.

At present, the evidence for empagliflozin in the treatment of HFpEF is still sparse 34. The prevalence of HFpEF increases with age 35. The increased risk of hospitalization for HF might be explained by the occurrence of multiple comorbidities in older adults with HFpEF 36. However, EMPEROR-Preserved did not include the total comorbidity burden as a covariate in their analysis 37. A prospective study was designed to assess the cognitive and physical function in consecutive frail older adults with diabetes and HFpEF, indicating empagliflozin improved Montreal cognitive assessment scores and physical impairment assessed by the 5-m gait speed test 38. EMPEROR-Preserved also confirmed that health status, as measured by the Kansas City Cardiomyopathy Questionnaire, improved at all time points when assessed (3, 8, and 12 months) for all domains (total symptom score, clinical summary score, and overall summary score^39^. Despite this, the benefits of empagliflozin were observed early and consistently. The nominal statistical significance was achieved for separation between the empagliflozin and the placebo arms by day 18 for time to cardiovascular death or hospitalization for HF, and this significance was sustained from there to the trial period 40. A similar pattern of early and sustained benefit was seen for health-related quality of life scores, the Kansas City Cardiomyopathy Questionnaire (total symptom score, clinical summary score, and overall summary score), and New York Heart Association functional class as well 41. Similarly, in EMPA-REG OUTCOME, the benefit of empagliflozin in reducing the risk of hospitalization for HF and cardiovascular death emerged within weeks after treatment initiation 42. The simplest explanation for such fast and short-term effects of empagliflozin in HFpEF is its diuretic effect 43. However, the diuretic effect of empagliflozin is different from loop diuretics in that empagliflozin caused a significant increase in 24-hour urine volume without an increase in urinary sodium and electrolyte-free water clearance 44 and a significant decrease in estimated plasma volume calculated by the Straus formula and estimated the extracellular volume determined by the body surface area 45. A pooled analysis of both the EMPEROR-Reduced and EMPEROR-Preserved trials indicated that the magnitude of the effect of empagliflozin on HF outcomes and health status was similar in patients with ejection fractions < 25% to < 65%, but it was attenuated in patients with an ejection fraction ≥ 65% 46, which may herald the recognition of a new phenotype characterized by supra-normal left ventricular ejection fraction 47.

We critically review several large clinical trials about empagliflozin, however, empagliflozin was not originally developed for HF and T2DM, the inclusion population is not particularly targeted in these trials, and any recommendation for the treatment of established HF and T2DM will be necessarily cautious. Additionally, well-designed clinical trials with long follow-ups may provide us with more valuable information.

## Abbreviations

Heart failure: HF
type 2 diabetes mellitus: T2DM
heart failure with reduced ejection fraction: HFrEF
heart failure with preserved ejection fraction: HFpEF
sodium-glucose co-transporter-2 inhibitor: SGLT-2i
